# 

**Published:** 1826-01

**Authors:** 


### Contents

OF

### No. 7. Vol. IV. (NEW SERIES) for JANUARY, 1826.

[*No.* 23, *ANALYTICAL SERIES.*]

Art. I.

Medical Researches on the Effects of Iodine in Bronchocele, Paralysis, Chorea, Scrofula, White Swellings, &c. By Alexander Manson, M.D. .. .. .. .. .. .. .. 1

II.

Practical Remarks on Lacerations of the Uterus and Vagina, with Cases. By Thomas M'Keever, M.D. .. .. .. .. .. 19

III.

Practical Remarks upon Indigestion, particularly as connected with Bilious and Nervous Affections of the Head and other Parts. By John Howship, Esq. .. .. .. .. .. .. 34

IV.

O[illegible]rvations on the Use of Colchicum Autumnale in the Treatment of Gout, &c. By Charles Scudamore, M.D.. .. .. 44

V.

Elements of the Anatomy of the Human Body in its sound State, with occasional Remarks on Physiology, Pathology, and Surgery. By Alexander Monro, M.D. F.R.S.E. &c. .. 52

VI.

1. Remarks on Irritative Fever, commonly called the Plymouth Dock-yard Disease. By John Butter, M.D. .. .. ..
2. Cases of the fatal Disease which occurred, during the last Autumn, in His Majesty's Dock-yard at Devonport. By E. Tripe, Esq. Surgeon.. .. .. .. .. .. .. ..
3. Official Report of Mr. Dryden respecting the Death of Dr. Bell, of Plymouth Dock-yard .. .. .. .. .. .. 66

VII.

Sketches of the most prevalent Diseases of India; including a Treatise on Epidemic Cholera, &c. By James Annesley, Esq. Madras Establishment.. .. .. .. .. .. .. .. 88

VIII.

The Lectures of Sir Astley Cooper, Bart. on the Principles and Practice of Surgery, with additional Notes and Cases. By Fred. Tyrrell, Esq

WI
ME 75 T

ART. 1. Inflammation and Irritation.. .. .. .. .. .. .. 98
2. Remarkable Case of Cessation of the Pulse in all Tangible Arteries for upwards of 40 hours, from the Introduction of a Bougie .. .. .. .. .. .. .. .. 102

IX.

The Works of Matthew Baillie, M.D. with an Account of his Life, collected from authentic Sources. By JAMES WARDROP, Surgeon to the King, &c. .. .. .. .. .. .. .. .. 115

X.

Collections from the unpublished Medical Writings of the late Dr. PARRY. Vol I. .. .. .... .. .. .. .. .. 124

XI.

Elements of Operative Midwifery, &c. By D. D. DAVIS, M.D. (Concluded from No. VI. of this Journal.).. .. .. .. 148

XII.

Quarterly Periscope

OF

PRACTICAL MEDICINE;

BEING

*THE SPIRIT OF THE MEDICAL JOURNALS,*

FOREIGN AND DOMESTIC.

I. PHYSIOLOGY.

1. M. Velpeau on Disorganization of the Medulla Oblongata, &c. without disturbance of the Nervous Functions. .. .. .. 178
2. Dr. Breyer on Venous Pulsation. .. .. .. .. .. .. .. 182
3. Dr. Deleau's curious Case of Hearing and Speech restored at the age of nine years. .. .. .. .. .. .. .. .. .. 183

II. PATHOLOGY.

1. M. Bayle on the new Doctrine of Mental Maladies .. .. 184

2. Mr. Howell on Phrenitis and Arteritis, with Remarks .. .. 192

3. Case of Pulmonary Phthisis cured.. .. .. .. .. .. .. 195

4. Laennec's Hospital Reports .. .. .. .. .. .. .. .. 196

   1. Fever... .. .. .. .. .. .. .. .. .. .. 197

   2. Acute Rheumatism .. .. .. .. .. .. .. 197

   3. Colica Pictonum—Peritonitis .. .. .. .. .. 198

   4. Phthisis .. .. .. .. .. .. .. .. .. .. 198

   5. Diseases of the Heart .. .. .. .. .. .. .. 199

   6. Aneurism .. .. .. .. .. .. .. .. .. 200

   7. Abdominal Tumours .. .. .. .. .. .. .. 201

5. M. Legarde's Case of Catalepsy .. .. .. .. .. .. 201

6. Memoir on the "Compact Œdema," or Induration of the Cellular Tissue of newly born Children. By Dr. Leger.. .. 204

7. Instances of Ulcers on the surface of the Brain .. .. .. 207

8. Hospital Reports from the Hotel Dieu .. .. .. .. .. 209
1. Intermittent Fevers .. .. .. .. .. .. .. .. 209
2. Arachnitis .. .. .. .. .. .. .. .. .. .. 210
3. Hæmoptysis.. .. .. .. .. .. .. .. .. .. 211
4. Pulmonic Inflammation .. .. .. .. .. .. .. 212

III. SURGERY.

1. Parallel between the French and German Surgery. By Dr. Ammon of Dresden, and Dr. Casper of Berlin. .. .. .. 213
2. Dr. Bishopp on Obliteration of Intestinal Canal in Hernia .. 231
3. Dr. Maitland's Case of Artificial Anus.. .. .. .. .. 233
4. Mr. Stevenson on the early removal of Cataract. .. .. .. 234
5. Mr. Sym's Case of Hydrocephalus tapped .. .. .. .. 236
6. Mr. Dewar on Amputation .. .. .. .. .. .. .. 237
7. Case of Rupture of the Ligamentum Patellæ.. .. .. .. 238
8. Mr. Mackenzie on Closure of the Pupil in Iritis .. .. .. 239
9. Dr. Corban's Case of Cranial Fracture.. .. .. .. .. 241
10. Mr. Braid on a peculiar ulcerous Affection .. .. .. .. 241
11. Remarks on Injuries of the Head .. .. .. .. .. .. 242
12. Dr. Briquet on Phlebectasia, or Varicose Veins .. .. .. 243

IV. THERAPŒIA.

1. Dr. Musgrave on Hepatic Ileus .. .. .. .. .. .. .. 248

2. Dr. Scott on diffuse inflammation of the Cellular Texture .. 255

3. Mr. Egan on Traumatic Tetanus in the Horse .. .. .. 257

4. Dr. Ainslie on Cholera Morbus .. .. .. .. .. .. 258

5. Dr. Williams on irregular Epilepsy .. .. .. .. .. .. 259

6. Dr. Venables on Chorea .. .. .. .. .. .. .. .. 260

7. M. Le Fevre on bleeding in Diabetes .. .. .. .. .. .. 260

8. Mr. Mackenzie on Croup .. .. .. .. .. .. .. .. 261

9. Mr. Barnard on pressure in Hydrocephalus.. .. .. .. 262

10. Inspector Tegart on Croton Oil .. .. .. .. .. .. 263

11. Hydrophobia.. .. .. .. .. .. .. .. .. .. .. 264

12. Mr. Brice's Stomach Pump .. .. .. .. .. .. .. 264

13. On the prevention of Hydrophobia .. .. .. .. .. .. 265

1. The Zurich Treatment.. .. .. .. .. .. .. .. 266

2. The Treatment of Wendt .. .. .. .. .. .. .. 266

14. Mr. Abraham's Case of Sanguineous Apoplexy .. .. .. .. 266

15. Successful treatment of Traumatic Tetanus. By Messrs. Manifold and Briggs .. .. .. .. .. .. .. .. .. 267

16. On the Treatment of Erysipelas .. .. .. .. .. .. .. 268

17. Mr. Wishart on Sympathetic Amaurosis .. .. .. .. .. 269

*Toxicology.*

1. Mismanaged case of Poisoning, M. Orfila present.. .. .. .. 271

2. Poisoning by Cantharides .. .. .. .. .. .. .. .. .. 272

V. MEDICAL JURISPRUDENCE.

1. Bye Law of the Royal College of Surgeons.—Dr. Armstrong's Letter .. .. .. .. .. .. .. .. .. .. .. .. .. 273

VI. MIDWIFERY.

1. Dr. Henry Davies on the Ergot of Rye .. .. .. .. .. .. 276
2. Dr. Bedel on Uterine Hæmorrhage.. .. .. .. .. .. .. 278
3. On the Transfusion of Blood .. .. .. .. .. .. .. .. 279
4. Gastrotomy in a Case of Extra-uterine Conception.. .. .. 280
5. On Vesico-vaginal Apertures .. .. .. .. .. .. .. .. 281

XIII.

ANALECTA MINORA.

1. On the Treatment of Colica Pictonum.. .. .. .. .. .. 282
2. Disgusting Depravation of Taste .. .. .. .. .. .. .. 282
3. Curious Professional Reclamations in France .. .. .. 283
4. Mr. Bush on the Cicatrization of large Sores .. .. .. 283
5. Mr. Doubleday on the Transfusion of Blood .. .. .. 283
6. Dr. Magee on Oil of Turpentine in Purpura.. .. .. .. 284
7. Case of Traumatic Tetanus cured.. .. .. .. .. .. .. 284
8. Forced Injections for Retention of Urine .. .. .. .. 284
9. National Compliments in Medicine .. .. .. .. .. .. 284
10. Incentives to Leeches .. .. .. .. .. .. .. .. .. 285
11. Case of Gastrotomy successful .. .. .. .. .. .. .. 285
12. Extra and Superfœtation .. .. .. .. .. .. .. .. 285
13. Tetanus cured by Caustic to the Spine.. .. .. .. .. 285
14. Remarkable Case of Thoracic Abscess mistaken .. .. .. 286
15. Death from a Worm in the Liver.. .. .. .. .. .. .. 287
16. Dr. Edwards on Electricity in Physiology .. .. .. .. 288

XIV

Bibliographical Record .. .. .. .. .. .. .. .. .. .. 289

XV.

EXTRA-LIMITES.

I. Mr. Weiss in answer to Dr. Davis.. .. .. .. .. .. .. 295

II. Mr Coles' Trusses.. .. .. .. .. .. .. .. .. .. .. 296

XVI.

INTELLIGENCE, CORRESPONDENCE, &c.

1. Prize Essays of the Edinburgh Royal Medical Society .. 299

2. Dr. Rutter's Reclamation .. .. .. .. .. .. .. .. 300

3. Obituary—Mr. J. B. Taylor, of Sunderland .. .. .. 300

4. Notices to various Correspondents .. .. .. .. .. .. 301

5. Oil of the Euphorbia Lathyrus—procurable in London .. 301

6. New Obstetric Society .. .. .. .. .. .. .. .. .. 302

7. Velpeau on the Spinal Marrow.. .. .. .. .. .. .. .. 302

8. Reproduction of the Crystalline Lens .. .. .. .. .. 303

9. Langier on Intestinal Concretions.. .. .. .. .. .. .. 303

10. Insensibility of the Retina .. .. .. .. .. .. .. .. 303

11. Sense of Smell not dependent on the Olfactory Nerves.. .. 303

12. Middlesex Infirmary—remodelled and enlarged .. .. .. 303

List of additional Subscribers .. .. .. .. .. .. .. .. 304

